# Measurement of Androgen and Estrogen Concentrations in Cord Blood: Accuracy, Biological Interpretation, and Applications to Understanding Human Behavioral Development

**DOI:** 10.3389/fendo.2014.00064

**Published:** 2014-05-02

**Authors:** Lauren P. Hollier, Jeffrey A. Keelan, Martha Hickey, Murray T. Maybery, Andrew J. O. Whitehouse

**Affiliations:** ^1^Telethon Kids Institute, University of Western Australia, Subiaco, WA, Australia; ^2^Neurocognitive Development Unit, School of Psychology, University of Western Australia, Crawley, WA, Australia; ^3^School of Women’s and Infants’ Health, University of Western Australia, Crawley, WA, Australia; ^4^Department of Obstetrics and Gynaecology, University of Melbourne, Parkville, VIC, Australia

**Keywords:** estrogens, androgens, cord blood, human development, prenatal

## Abstract

Accurately measuring hormone exposure during prenatal life presents a methodological challenge and there is currently no “gold standard” approach. Ideally, circulating fetal hormone levels would be measured at repeated time points during pregnancy. However, it is not currently possible to obtain fetal blood samples without significant risk to the fetus, and therefore surrogate markers of fetal hormone levels must be utilized. Umbilical cord blood can be readily obtained at birth and largely reflects fetal circulation in late gestation. This review examines the accuracy and biological interpretation of the measurement of androgens and estrogens in cord blood. The use of cord blood hormones to understand and investigate human development is then discussed.

Prenatal exposure to sex steroids has long been posited to influence human development (1, 2). There are a number of biological differences between males and females, including height, weight, internal reproductive anatomy, and external genitalia. Sex differences have also been observed in neuroanatomy and neurochemistry (3). In addition to biological differences, males and females differ in a number of behavioral and cognitive aspects. Sex differences have been found for aggression (4, 5), childhood play (6), visuospatial ability (7, 8), and verbal ability (9–11). However, the examination of the relationship between these sex differences and sex steroid exposure has been limited due to inherent difficulties in providing accurate and reliable measurements of hormone exposure during prenatal life.

Findings from animal models illustrate that exposure to sex steroids during critical periods of gestational development can have a significant long-term impact (12). However, due to the many developmental differences between species (e.g., duration of pregnancy, maturity at birth, and susceptibility to a variety of environmental conditions), it is difficult to extrapolate animal model findings to human development. Human brain and tissue samples cannot realistically be collected for research purposes during normal pregnancy, and it is not ethical to manipulate hormone levels in human fetuses. Therefore, surrogate markers of fetal hormone levels, such as the second-to-fourth digit (2D:4D) ratio or the examination or clinical populations exposed to atypical hormone levels (e.g., congenital adrenal hyperplasia), have been used to investigate the relationship between sex steroids and human development. However, there is doubt as to whether the 2D:4D ratio is a reliable proxy measure of fetal testosterone levels (13, 14) and it is difficult to extrapolate findings from clinical populations to typical human development.

Alternatively, fetal hormones can be measured in amniotic fluid from the second trimester of pregnancy onward. Amniotic samples provide an approximation of circulating fetal hormones by gaging the hormone levels that have entered the amniotic fluid via fetal urination or diffusion through fetal skin (15). While amniotic hormones are thought to relate to hormone levels in fetal blood, the strength of the relationship remains unclear. In addition, the sampling procedure (amniocentesis) is generally only performed in high-risk pregnancies and could not ethically be performed in low risk pregnancies solely for research purposes.

Currently, there is no “gold standard” approach to the measurement of prenatal hormone exposure (16). In response to the methodological difficulties, researchers have sought alternative means through which prenatal hormone levels can be approximated. Umbilical cord plasma collected at birth allows for the collection of large representative samples and analysis of archived samples. The current review will examine the biological interpretation of cord blood hormones, issues with assay, and steroid comparisons in cord blood sex steroids and applications of this measurement approach to understanding human development.

## Biological Interpretation of Cord Blood Hormones

Pregnancy represents a unique phase of human life where circulating hormone concentrations are derived from maternal, placental, and fetal origins. The developing fetal endocrine environment is a function of gonadal, adrenal, and placental biosynthesis, metabolism, and biodistribution, modulated by protein binding, biological activity, and receptor affinity (17). The placenta is a highly steroidogenic organ responsible for the production of large amounts of free androgens and estrogens from fetal adrenal and gonadal precursors (18, 19). The placenta is also a very active metabolic organ with respect to phase II metabolism (conjugation). While steroids are lipophilic and cross the placenta in both directions, most of them are metabolized by the placenta *en route* (20). Fetal blood leaves the placenta (enriched with placental steroid metabolites and some maternal steroids) via the umbilical vein (UV) and returns from the fetus to the placenta via the umbilical artery [UA; (21)].

Umbilical cord blood is typically collected after delivery near term, and so cord plasma or serum hormone concentrations are thought to reflect the levels in the fetal circulation at late gestation (22). Umbilical cord blood samples contain approximately equal amounts of venous (UV) and arterial (UA) components, although the relative proportions are usually not known precisely and are not controlled for. Nevertheless, despite the differences in UV and UA steroid concentrations, they are strongly correlated (19). Ideally, fetal blood would be sampled earlier in pregnancy, during critical periods of development, but this is not feasible for ethical reasons. Hormone concentrations from maternal blood samples collected during normal pregnancy have been suggested as a possible approximation of circulating fetal concentrations (16). However, several studies have shown that maternal sex steroid concentrations do not reflect those in fetal circulation and the relationships are weak (23–25). Amniotic fluid sampled during mid gestation provides a proxy measure of blood hormone concentration in the fetus (15). However, the exact relationship between hormone concentrations in amniotic-fluid and circulating fetal concentrations remains unclear. Furthermore, due to the invasive nature of amniocentesis it is restricted to high-risk pregnancies. Currently, cord blood is the only practical means of assessing fetal hormone levels during a typical pregnancy (26, 27).

It is important to note that the measurement of umbilical cord sex steroids may be affected by a number of obstetric and maternal factors. Fetal adrenal steroid production changes with gestational age and labor, while levels of steroid-metabolizing enzymes in the placenta are regulated by factors known to be associated with labor and delivery such as glucocorticoids, pro-inflammatory cytokines, and exposure to reactive oxygen species (18). Hence, it is highly likely that factors such as prematurity, labor onset, placental weight, intrauterine infection, and pre-eclampsia could influence umbilical cord androgen levels, although the nature and extent of their influence have not yet been fully determined. However, these relationships have recently been investigated in a large unselected birth cohort; it was found that the presence and duration of labor and gestational age at delivery significantly impact upon androgen and estrogen concentrations in cord blood (22, 28). In addition, birth weight and the presence of ante-partum hemorrhage or pre-eclampsia significantly impact cord estrogen levels (28). Some studies also suggest that smoking in pregnancy may increase circulating postnatal testosterone and cortical concentrations and reduce estriol concentrations (29, 30), although these relationships were not observed in the large cohort study of Hickey et al. (28). Other maternal factors such as ethnicity, age, and parity should also be controlled for when examining cord blood hormones (22).

Another important consideration is that the major circulating sex steroids (i.e., testosterone, estradiol, and estrone) are bound to proteins such as sex hormone binding globulin (SHBG) and albumin, which impacts upon their bioactivity (31). Albumin and SHBG values vary according to gestational age at delivery and onset of labor (22, 26). Therefore, accurate evaluation and interpretation of prenatal sex steroid concentrations requires adjustment for pregnancy concentrations of albumin and SHBG in order to determine the biologically active fraction (22, 26).

Normal values for sex steroid concentrations have been derived from birth cohorts and consistently demonstrate that testosterone concentrations are higher in males compared to females (25, 32, 33). Umbilical cord blood androgens are likely to reflect the fetal androgen environment during late gestation. However, a major limitation of the use of cord blood is that it is possible that androgen influences on development may occur earlier in gestation (34).

Unlike androgens, umbilical cord estrogen concentrations do not consistently differ significantly between males and females. Studies have reported inconsistent results including no sex differences (24, 25, 28, 32, 33, 35), higher estrogen concentrations in females (36), and higher estrogen concentrations in males (16, 37). The lack of consistent sex differences in estrogen concentrations is biologically and clinically significant. Sex differences in estrogen exposure and in the ratio of estrogen to testosterone have been postulated to be responsible for a variety of sexually dimorphic neurodevelopmental and behavioral characteristics including sexual orientation (38), reproductive function (12), and cardiovascular disease (39).

It is clear from the literature reviewed that the collection of cord blood plasma is useful as a measure of late gestation circulating fetal hormones. However, it is important when measuring cord hormone concentrations, to adjust for obstetric and maternal confounding factors as well as to calculate the biological active fractions of the sex steroids in order to derive valid conclusions.

## Issues Related to the Determination of Steroid Concentrations in Cord Blood

Cord serum or plasma represents a particularly challenging medium to analyze as it has an unusual steroid profile due to the unique combination of placental and fetal steroid biosynthesis and metabolism [conjugation and deconjugation; (40, 41)]. Therefore, assays require particularly careful validation to ensure that the results are accurate. Due to the presence of a variety of steroid isomers in cord blood with almost identical structures and fragmentation patterns, efficient chromatographic discrimination of analytes during analysis can be particularly important – even when using mass spectrometry as the detection methodology. Most published studies have used radioimmunoassay (RIA) to measure concentrations of estrogens and androgens in cord blood (16, 24, 25, 37, 42–55). Organic solvent extraction has been employed in most studies to remove interfering factors in order to improve specificity, accuracy, and sensitivity. A few studies have used additional column purification techniques (24, 25, 53) to address concerns regarding the accuracy of RIAs for the measurement of low concentrations of steroids, in particular, testosterone (56, 57). However, while the assays are sometimes tested and validated for use in female or pediatric samples, their suitability for umbilical cord blood analysis is usually assumed and invariably not adequately tested.

**Figure 1:**
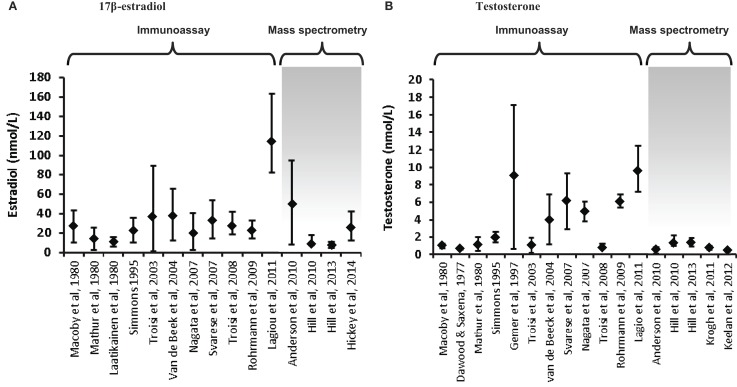
**Variations in umbilical cord blood steroid concentrations according to mode of assay**. Steroid concentrations in cord blood are displayed as mean ± standard deviation or median and interquartile range, depending on the data available. **(A)** 17β-estradiol concentrations in cord blood (males and females combined) are displayed from 15 studies. The first 11 utilized immunoassay (predominantly RIA); the remaining 4 used LC–MS/MS, apart from the studies by Hill et al. (41), which used GC–MS. **(B)** Testosterone concentrations in cord blood (males or mixed male/female samples). Of the 17 studies included, the final 5 employed mass spectrometry. The last study displayed on each chart has the largest samples size (*n* = 860) and extensive assay validation for cord blood. The steroid values from the studies by Hill et al. (41) are the mean of the published umbilical artery and vein values. The figure demonstrates the influence of assay characteristics on sex steroid values, although the lack of assay specificity is much more significant for measurement of testosterone compared to estradiol.

Increasing awareness of the limitations of RIA for the measurement of low concentrations of sex steroids (56, 58, 59) has led to the adoption of mass spectrometry as the preferred methodology for the measurement of circulating testosterone levels in women and children, and reported concentrations are consistently lower than those derived by RIA (58, 60–63). A comparison of estradiol and testosterone concentrations in cord blood reported over the past few decades in numerous studies reveals the significant impact of assay methodology on the results obtained, and thus the robustness of the data and any conclusions drawn from it (Figure 1). Firstly, it is clear that some assays have reported far higher ranges of these hormones than others, even for the same type of assay; this is mostly apparent in the studies that have employed RIA and raises real concerns around the validity of some of these studies. Secondly, it is clear that much lower values for testosterone are consistently reported in studies that have employed mass spectrometry (either LC–MS/MS or GC–MS) compared to immunoassay-derived values. On the other hand, estradiol levels are overall not dramatically different between RIA and mass spectrometry-based studies [with the exception of the study by Lagiou et al. (46)]. Closer inspection of the immunoassay studies, which reported the lowest testosterone values reveals that these assays employed column purification to help remove interfering cross-reacting steroids (24, 25, 53). Nevertheless, these studies still reported values about 40% higher than those reported by the largest study to date which employed an extensively validated and internally controlled LC–MS/MS assay (22), suggesting that chromatographic treatment was not completely effective in removing all cross-reacting compounds prior to RIA analysis.

Even within the mass-spectrometry studies, there are sometimes quite marked variations in reported values (22, 28, 35, 36, 41, 60). This can reflect variations in internal standardization and extraction efficiency, with some studies measuring multiple hormones and conjugates without controlling for extraction efficiency of each specific analyte (35, 41). Inadequate chromatographic separation of similar steroid species prior to mass spectrometry is also an important source of variation. However, there is also a great deal of variation in the nature of the specimens analyzed in the studies, such that differences in mode of collection, ethnicity, gestational age, labor onset and duration, rates of pregnancy complications, twinning, and sample number/power are all likely to have a major effect on the values obtained (22, 28).

## Applications of Cord Blood to Understanding Human Development

The literature reviewed provides general support for the use of cord blood measurements to approximate prenatal sex steroid concentrations, although limitations regarding confounding by mode of delivery and method of analysis need to be considered in the interpretation. The remainder of this review will examine extant applications of cord blood to understanding human behavioral development.

To date, two research groups have used cord blood to examine the relationship between human development and exposure to prenatal hormones (see Table 1). Jacklin and colleagues from Stanford University examined a group of participants combined from three longitudinal cohorts. Cohort one comprised infants born at a university hospital during July and August 1973 (40 males, 35 females). The infants in cohorts two and three were born at a nearby general hospital during January–March 1974 (32 males, 42 females) and August–November 1974 (53 males, 54 females), respectively. For offspring to be included in the study, there needed to be no complications of pregnancy or delivery and a 5-min Apgar score of 7 or above. Umbilical cord blood (venous and arterial) was collected at birth and androstenedione, testosterone, estrone, estradiol, and progesterone concentrations were determined by RIA. Cord testosterone concentrations were significantly higher in males than females. No sex differences were observed for the androstenedione, estrone, estradiol, or progesterone levels (33).

**Table 1:** **Summary of the studies examining the relationship between cord hormone concentrations and human development**.

Study	Number of participants	Age of participants	Measures			Findings
			Hormones	Assay technique	Outcome measure	
Jacklin et al. (64)	84 males; 78 females	6, 9, 12, and 18 months	Androstenedione; testosterone; estrone; estradiol; progesterone	RIA	Timidity	**Males:** significant negative relationship with testosterone and progesterone, positive relationship with estradiol
						**Females:** no significant relationship
Jacklin et al. (65)	127 children;	Birth, 3, 6, 9, 12, 18, and 33 months	Androstenedione; testosterone; estrone; estradiol; progesterone	RIA	Muscular strength	**Males:** significant negative relationship with androstenedione, significant positive relationship with progesterone
						**Females:** significant negative relationship with androstenedione and progesterone
Jacklin et al. (66)	53 males; 43 females	6 years	Androstenedione; testosterone; estrone; estradiol; progesterone	RIA	Reading; Numeracy; Listening; Spatial ability	**Males:** no significant relationship
						**Females:** significant inverse relationship between testosterone and androstenedione and spatial ability
Hollier et al. (67)	224 males; 199 females	2 years	Testosterone	LC–MS	Vocabulary	**Males:** significant inverse relationship
						**Females:** no significant relationship
Whitehouse et al. (68)	372 males; 395 females	1–3 years	Testosterone	LC–MS	Language delay	**Males:** significant inverse relationship
						**Females:** no significant relationship
Farrant et al. (69)	235 males; 232 females	1 and 5 years	Testosterone	LC–MS	Socio-emotional engagement; Vocabulary;	**Males:** no significant relationship after control for covariates
						**Females:** no significant relationship
Whitehouse et al. (70)	184 males; 190 females	19–20 years	Testosterone	LC–MS	Autism Quotient	**Males:** no significant relationship
						**Females:** no significant relationship
Robinson et al. (71)	429 males; 430 females	2, 5, 8, and 10 years	Testosterone	LC–MS	Child Behavior Checklist	**Males:** negative relationship between testosterone and attention problems at ages 5, 8, and 10 years
						**Females:** negative association between testosterone and withdrawal symptoms at age 5

Timidity was measured through observation in 162 offspring (84 males, 78 females) at 6, 9, 12, and 18 months (64). No sex difference was found for the timidity measure. For boys, testosterone and progesterone levels were significant negative predictors of timidity, and estradiol levels were a significant positive predictor. No significant relationships were observed between cord hormones concentrations and timidity for girls.

Jacklin et al. (65) investigated the relationship between umbilical cord hormones and muscular strength measured at birth, 3, 6, 9, 12, 18, and 33 months in 127 children. On average, males had greater strength scores than females. A significant negative relationship was found between androstenedione and strength in males and females. However, this relationship was found to be reversed in one of the three cohorts, indicating the relationship is not stable. A significant sex by progesterone interaction was found in all three cohorts, where higher progesterone levels were related to greater strength for males, but to lower strength for females.

The final study by Jacklin and colleagues examined cognitive abilities in a subsample of 96 children (53 males, 43 females) when they were 6 years old. No significant sex differences were found for cognitive ability. A significant inverse relationship was found between cord androgens (testosterone and androstenedione) and spatial ability in girls. No significant associations were found for boys (66).

The data yielded by Jacklin and colleagues indicate that the direction of the association between cord blood hormones and outcome measures may be different between males and females. For boys, cord progesterone concentrations were positively associated with strength in early childhood and negatively related to timidity. For girls, cord progesterone was negatively associated with early childhood strength and cord androgens (testosterone and androstenedione) were inversely related to spatial ability. It should be noted that Jacklin and colleagues measured cord blood hormone concentrations by RIA following extraction and purification. In addition, they used total hormone concentrations, rather than the biologically active proportion. However, the hormone levels reported were similar to a number of studies using mass spectrometry (see Figure 1) and their data appear reliable.

The second research group utilized data from the Western Australian Pregnancy Cohort (Raine) study. Between May 1989 and November 1991, 2900 pregnant women were recruited from King Edward Memorial Hospital or nearby private practices. To be included in the study, pregnant women had to have sufficient English language skills, a gestational age between 16 and 18 weeks, an expectation to give birth at King Edward Memorial Hospital Perth and an intention to remain within the state so as to enable follow-up testing. Parents provided written informed consent to participate at each follow-up. Approximately half of the cohort (*n* = 1415) was randomly allocated to an intensive investigation group, and within this group, mixed (venous and arterial) umbilical cord blood was randomly collected from 861 live deliveries. Testosterone, androstenedione, dehydroepiandrosterone (DHEA), estrone, estradiol, estriol, and estetrol concentrations were estimated using LC–MS/MS. The bioavailable proportion of testosterone was calculated, representing the fraction of total testosterone either free (unsequestered by SHBG) or bound to serum albumin. Males had significantly higher levels of cord testosterone than females, while females had significantly higher DHEA concentrations than males (22). There were no significant sex differences for androstenedione, estrone, estradiol, estriol, or estetrol (22, 28).

When offspring were 2 years of age, parents of 426 children (224 male; 199 female) completed the Language Development Survey [LDS; (72)]. Consistent with extensive published literature (73–75), it was found that on average boys had a smaller expressive vocabulary than girls at 2 years of age. In addition, an inverse relationship was found between cord blood testosterone and expressive vocabulary, where higher levels of cord blood testosterone predicted lower vocabulary size in the boys. No relationship was observed in girls (67).

When the offspring were 1, 2, and 3 years of age, parents of 767 children (395 males; 372 females) completed the 12, 24, and 36 month versions of the Infant Monitoring Questionnaire [IMQ; (76)]. The IMQ is a parent report checklist, designed to screen for delayed child development during the early years. Cut-off scores are provided for each scale at each age to indicate a “clinically significant” delay in the development of that particular skill. It was found that males were between two and three times more likely than females to experience language delay at 1, 2, and 3 years of age. In addition, there was a significant association between cord blood testosterone and the rate of language delay in both males and females (70). In line with Hollier et al. (67), it was found that higher levels of cord blood testosterone increased the risk of language delay in males. Interestingly, for females, higher levels of cord blood testosterone were found to be protective for language delay. No significant relationship was observed between the other IMQ scales and cord blood testosterone. The non-significant finding on the Personal–Social scale of the IMQ is in contrast with an inverse association reported by Knickmeyer et al. (77) between amniotic-fluid testosterone levels and parent-reported social skills in their sample of children at 4 years.

Farrant et al. (69) further explored the relationship between cord blood testosterone, socio-emotional engagement, and language development in a subset of 467 children from the Raine sample (235 males, 232 females). Socio-emotional engagement was assessed when the children were 1-year-old using a 14-item Australian revision of the Toddler Temperament Scale (78). Vocabulary was measured using the Peabody Picture Vocabulary Test – Revised [PPVT-R; (79)], when the children were 5 years old. It was found that cord blood testosterone was significantly negatively correlated with socio-emotional engagement and vocabulary in boys. In addition, for boys, socio-emotional engagement was found to completely mediate the relationship between fetal testosterone and vocabulary development. However, when various covariates were included (e.g., maternal age and education, parity, and parent–child book reading) neither fetal testosterone nor socio-emotional engagement were significant predictors of vocabulary development. No significant relationships were observed for girls.

To further extend, the research examining the link between socio-emotional engagement and cord blood testosterone, Whitehouse et al. (68) examined the relationship between cord testosterone and autistic-like traits in adulthood. When the offspring were 19–20 years old 184 males and 190 females completed the Autism-spectrum Quotient [AQ; (80)]. The AQ is a self-report questionnaire, where individuals are provided with 50 statements and asked to indicate on a four-point scale how well that statement applies to them. No significant relationship was found between cord blood testosterone concentrations and AQ scores for either males or females. This finding is in contrast with results from the Cambridge Fetal Testosterone Project, which include reports of significant associations between amniotic testosterone concentrations and a range of autistic-like traits during early (77, 81, 82) and middle (83) childhood.

The findings from Whitehouse et al. (70), Farrant et al. (69), and Whitehouse et al. (68) indicate there may be no relationship between socio-emotional development and cord blood testosterone at birth. These findings contrast with studies of mid-pregnancy amniotic-fluid steroids, which indicate that socio-emotional development may be related to testosterone exposure during the earlier stages of gestation. The cord blood studies have the advantage that they were conducted on a very large cohort of typically developing infants, although steroid levels were measured at delivery, many weeks after the period expected to be critical for steroid-modulated neurodevelopment. The amniotic-fluid studies, on the other hand, were on relatively small numbers of selected pregnancies with elevated risk of genetic abnormalities, although samples were taken closer to the expected steroid-sensitive developmental window.

Finally, Robinson et al. (71) investigated the relationship between cord blood testosterone and internalizing and externalizing behavior in childhood for the Raine sample. Externalizing and internalizing behaviors were measured when the children (429 males; 430 females) were 2, 5, 8, and 10 years of ages using the Child Behavior Checklist [CBCL; (84, 85)]. The CBCL is a parent-rated questionnaire that includes a Total Behavior Problem scale, an Externalizing Behavior scale (e.g., antisocial or under-controlled behavior), and an Internalizing Behavior scale (e.g., inhibited or over-controlled behavior). In addition, there are eight subscales: withdrawn behavior, somatic complaints, anxiety/depression, delinquency, aggression, social thought, and attention problems. No significant relationships were found between cord blood testosterone and the CBCL total, internalizing or externalizing scales at all ages. When examining the individual subscales, a negative relationship was found between cord testosterone and attention problems for boys at ages 5, 8, and 10 years. For girls, there was a negative association between cord testosterone and withdrawal symptoms at age five. The findings from Robinson et al. (71) did not demonstrate a consistent relationship between fetal testosterone exposure and behavioral difficulties in childhood. However, significant relationships were observed for particular behaviors, which suggest there may be links between fetal testosterone exposure and behavioral development. Further research is warranted to more thoroughly understand the relationship between fetal testosterone exposure and behavioral difficulties in childhood.

In summary, significant negative relationships have been consistently observed between cord blood testosterone and early language development. However, relationships between cord testosterone and other forms of development are still unclear. Some studies indicate that high fetal testosterone exposure is related to more male-typical behavior, while others have found that high fetal testosterone is associated with less male-typical behavior. It is important to note that the analysis of the Raine study samples utilized the more accurate LC–MS/MS method, and calculated the bioavailable proportion of testosterone, both of which add strength to the quality of the data. The studies by Jacklin and colleagues, on the other hand, used a robust RIA but were limited to the use of total hormone concentrations uncorrected for protein binding. To date, only a few studies have been conducted in this area and relationships between cord testosterone and childhood development should be investigated more thoroughly in the future. Furthermore, only Jacklin and colleagues have investigated relationships between cord blood estrogens and early development. Cord estrogen levels have been measured in the Western Australian Pregnancy Cohort; however, the relationship between cord estrogen concentrations and human development has not yet been examined in the cohort.

## Conclusion

The purpose of the current review was to examine the use of cord blood to measure androgen and estrogen concentrations, and the applicability of this method to understanding human development. From the literature reviewed, it is apparent that cord blood is useful in providing direct measurement of late gestational androgen and estrogen concentrations. However, fetal steroid levels around the time of birth are influenced by obstetric and perinatal factors. It is essential for the accuracy of analyses to take these factors into account when examining associations between cord steroid levels and biological endpoints.

Furthermore, it is important that research using cord blood to measure sex steroids employs properly validated assays. This is to ensure that cross-reacting substances are not measured in error, which is particularly important for the measurement of testosterone concentrations. Most studies to date have used RIA, which has recognized limitations for the measurement of low sex steroid concentrations (56, 58, 59). Mass spectrometry is currently the preferred method for the measurement of circulating hormones in cord blood.

Relatively little research has been performed on cord blood to examine the relationship between fetal hormone exposure and human neurodevelopment. Most of the research in this area has focused on testosterone concentrations. A significant negative relationship between cord testosterone and early language development has been consistently observed (67, 70). However, the relationships between cord testosterone and other aspects of development are still unclear. One of the main criticisms of the use of cord blood to examine the hormonal influences of human development is that hormone effects may occur earlier in gestation. The strength of the relationship between fetal plasma levels at birth and those in early to mid pregnancy is not known. One advantage of cord blood is that it provides a measure of hormone exposure later in gestational development. This is of particular importance given that animal studies have found that the effects of hormones on development are not restricted to the first two trimesters (12, 86). Examining the congruence between hormones obtained during an earlier period of gestation (e.g., via amniotic fluid) and hormones obtained from cord blood is an urgent priority for this field of research.

To date, only Jacklin and colleagues have examined the relationship between cord blood estrogens and human development. However, cord blood hormone concentrations were measured using RIA (64–66). In addition, adjustments for protein binding were not taken into account. Therefore, it is difficult to draw definitive conclusions from their findings. More research needs to be conducted to examine the relationship between human development and cord estrogen concentrations. Furthermore, based on conflicting evidence of sex differences in cord estrogen concentrations (16, 24, 25, 28, 32, 33, 35–37), it has been suggested that the ratio of androgens to estrogens may underlie sex-associated developmental outcomes, rather than absolute concentrations. Future research should investigate possible relationships between the androgen to estrogen ratio and aspects of human development.

In conclusion, the collection of cord blood is currently the most practical means of approximating circulating fetal hormones in normal pregnancy. The use of cord blood to examine the relationship between fetal hormones and human development is promising, but requires validation and further investigation. It is important that future research in this area uses properly validated assays to determine hormone concentrations and takes into account any possible confounding factors. The results of studies that do not take these steps may lack accuracy and valid interpretation.

## Conflict of Interest Statement

The authors declare that the research was conducted in the absence of any commercial or financial relationships that could be construed as a potential conflict of interest.

## References

[1] Collaer ML Hines M Human behavioral sex differences: a role for gonadal hormones during early development? Psychol Bull (1995) 118(1):55–107 10.1037/0033-2909.118.1.55 7644606

[2] Finegan J-A Bartleman B Wong P A window for the study of prenatal sex hormone influences on postnatal development. J Genet Psychol (1989) 150(1):101–12 10.1080/00221325.1989.9914580 2496195

[3] Ngun TC Ghahramani N Sánchez FJ Bocklandt S Vilain E The genetics of sex differences in brain and behavior. Front Neuroendocrinol (2011) 32(2):224–46 10.1016/j.yfrne.2010.10.001 20951723 PMC3030621

[4] Bettencourt B Miller N Gender differences in aggression as a function of provocation: a meta-analysis. Psychol Bull (1996) 119(3):422 10.1037/0033-2909.119.3.422 8668747

[5] McIntyre MH Barrett ES McDermott R Johnson DDP Cowden J Rosen SP Finger length ratio (2D:4D) and sex differences in aggression during a simulated war game. Pers Individ Dif (2007) 42(4):755–64 10.1016/j.paid.2006.08.009

[6] Hines M Brain Gender. New York: Oxford University Press (2004).

[7] Collaer ML Nelson JD Large visuospatial sex difference in line judgment: possible role of attentional factors. Brain Cogn (2002) 49(1):1–12 10.1006/brcg.2001.1321 12027388

[8] Voyer D Voyer S Bryden MP Magnitude of sex differences in spatial abilities: a meta-analysis and consideration of critical variables. Psychol Bull (1995) 117(2):250 10.1037/0033-2909.117.2.250 7724690

[9] Herlitz A Airaksinen E Nordström E Sex differences in episodic memory: the impact of verbal and visuospatial ability. Neuropsychology (1999) 13(4):590 10.1037/0894-4105.13.4.590 10527068

[10] Lewin C Wolgers G Herlitz A Sex differences favoring women in verbal but not in visuospatial episodic memory. Neuropsychology (2001) 15(2):165 10.1037/0894-4105.15.2.165 11324860

[11] Sherwin BB Estrogen and cognitive functioning in women. Endocr Rev (2003) 24(2):133–51 10.1210/er.2001-0016 12700177

[12] Zambrano E Guzmán C Rodríguez-González GL Durand-Carbajal M Nathanielsz PW Fetal programming of sexual development and reproductive function. Mol Cell Endocrinol (2014) 382(1):538–49 10.1016/j.mce.2013.09.008 24045010

[13] Dean A Sharpe RM Anogenital distance or digit length ratio as measures of fetal androgen exposure: relationship to male reproductive development and its disorders. J Clin Endocrinol Metab (2013) 98(6):2230–8 10.1210/jc.2012-4057 23569219

[14] Knickmeyer RC Woolson S Hamer RM Konneker T Gilmore JH 2D:4D ratios in the first 2 years of life: stability and relation to testosterone exposure and sensitivity. Horm Behav (2011) 60(3):256–63 10.1016/j.yhbeh.2011.05.009 21664359 PMC3143220

[15] Nagami M McDonough P Ellegood J Mahesh V Maternal and amniotic fluid steroids throughout human pregnancy. Am J Obstet Gynecol (1979) 134:674–80 463959 10.1016/0002-9378(79)90649-5

[16] van de Beek C Thijssen JHH Cohen-Kettenis PT van Goozen SHM Buitelaar JK Relationships between sex hormones assessed in amniotic fluid, and maternal and umbilical cord serum: what is the best source of information to investigate the effects of fetal hormonal exposure? Horm Behav (2004) 46(5):663–9 10.1016/j.yhbeh.2004.06.010 15555509

[17] Kuijper EAM Ket JCF Caanen MR Lambalk CB Reproductive hormone concentrations in pregnancy and neonates: a systematic review. Reprod Biomed Online (2013) 27(1):33–63 10.1016/j.rbmo.2013.03.009 23669015

[18] Albrecht ED Pepe GJ Placental steroid hormone biosynthesis in primate pregnancy. Endocr Rev (1990) 11(1):124–50 10.1210/edrv-11-1-124 2180685

[19] Pašková A Parízek A Hill M Velíková M Kubátová J Dušková M Steroid metabolome in the umbilical cord: is it necessary to differentiate between arterial and venous blood? Physiol Res (2014) 63(1):115–26 24182340 10.33549/physiolres.932624

[20] Reis FM Florio P Cobellis L Luisi S Severi FM Bocchi C Human placenta as a source of neuroendocrine factors. Biol Neonate (2001) 79(3–4):150–6 10.1159/000047083 11275643

[21] Ishimoto H Jaffe RB Development and function of the human fetal adrenal cortex: a key component in the feto-placental unit. Endocr Rev (2011) 32(3):317–55 10.1210/er.2010-0001 21051591 PMC3365797

[22] Keelan JA Mattes E Tan H Dinan A Newnham JP Whitehouse AJO Androgen concentrations in umbilical cord blood and their association with maternal, fetal and obstetric factors. PLoS One (2012) 7(8):e42827 10.1371/journal.pone.0042827 22916165 PMC3423422

[23] Cohen-Bendahan CCC van Goozen SHM Buitelaar JK Cohen-Kettenis PT Maternal serum steroid levels are unrelated to fetal sex: a study in twin pregnancies. Twin Res Hum Genet (2005) 8:173–7 10.1375/twin.8.2.173 15901481

[24] Troisi R Potischman N Roberts J Siiteri P Daftary A Sims C Associations of maternal and umbilical cord hormone concentrations with maternal, gestational and neonatal factors (United States). Cancer Causes Control (2003) 14(4):347–55 10.1023/A:1023934518975 12846366

[25] Troisi R Potischman N Roberts JM Harger G Markovic N Cole B Correlation of serum hormone concentrations in maternal and umbilical cord samples. Cancer Epidemiol Biomarkers Prev (2003) 12(5):452–6 12750241

[26] Hickey M Sloboda DM Atkinson HC Doherty DA Franks S Norman RJ The relationship between maternal and umbilical cord androgen levels and polycystic ovary syndrome in adolescence: a prospective cohort study. J Clin Endocrinol Metab (2009) 94(10):3714–20 10.1210/jc.2009-0544 19567524

[27] Sloboda DM Hickey M Hart R Reproduction in females: the role of the early life environment. Hum Reprod Update (2011) 17(2):210–27 10.1093/humupd/dmq048 20961922

[28] Hickey M Hart R Keelan JA The relationship between umbilical cord estrogens and perinatal characteristics. Cancer Epidemiol Biomarkers Prev (2014). 10.1158/1055-9965.EPI-13-1321 24636976

[29] Smith LM Cloak CC Poland RE Torday J Ross MG Prenatal nicotine increases testosterone levels in the fetus and female offspring. Nicotine Tob Res (2003) 5(3):369–74 10.1080/146222031000094196 12791533

[30] Varvarigou AA Liatsis SG Vassilakos P Decavalas G Beratis NG Effect of maternal smoking on cord blood estriol, placental lactogen, chorionic gonadotropin, FSH, LH, and cortisol. J Perinat Med (2009) 37(4):364–9 10.1515/JPM.2009.028 19290844

[31] Pardridge WM Serum bioavailability of sex steroid hormones. Clin Endocrinol Metab (1986) 15(2):259–78 10.1016/S0300-595X(86)80024-X 3521955

[32] Herruzo AJ Mozas J Alarcón JL López JM Molina R Molto L Sex differences in serum hormone levels in umbilical vein blood. Int J Gynecol Obstet (1993) 41(1):37–41 10.1016/0020-7292(93)90152-M 8098294

[33] Maccoby EE Doering CH Jacklin CN Kraemer H Concentrations of sex hormones in umbilical-cord blood: their relation to sex and birth order of infants. Child Dev (1979) 50(3):632–42 10.2307/1128928 498842

[34] Beek C Goozen SM Buitelaar J Cohen-Kettenis P Prenatal sex hormones (maternal and amniotic fluid) and gender-related play behavior in 13-month-old infants. Arch Sex Behav (2009) 38(1):6–15 10.1007/s10508-007-9291-z 18080735

[35] Hill M Pašková A Kanceva R Velíková M Kubátová J Kancheva L Steroid profiling in pregnancy: a focus on the human fetus. J Steroid Biochem Mol Biol (2014) 139(0):201–22 10.1016/j.jsbmb.2013.03.008 23583279

[36] Anderson H Fogel N Grebe SK Singh RJ Taylor RL Dunaif A Infants of women with polycystic ovary syndrome have lower cord blood androstenedione and estradiol levels. J Clin Endocrinol Metab (2010) 95(5):2180–6 10.1210/jc.2009-2651 20228162 PMC2869542

[37] Simmons D France JT Keelan JA Song L Knox BS Sex differences in umbilical cord serum levels of inhibin, testosterone, oestradiol dehydroepiandrosterone sulphate, and sex hormone-binding globulin in human term neonates. Neonatology (1994) 65(5):287–94 10.1159/000244074 8054396

[38] Balthazart J Minireview: hormones and human sexual orientation. Endocrinology (2011) 152(8):2937–47 10.1210/en.2011-0277 21693676 PMC3138231

[39] Sathishkumar K Elkins R Yallampalli U Balakrishnan M Yallampalli C Fetal programming of adult hypertension in female rat offspring exposed to androgens in utero. Early Hum Dev (2011) 87(6):407–14 10.1016/j.earlhumdev.2011.03.001 21450421 PMC3093104

[40] Clifton VL Bisits A Zarzycki PK Characterization of human fetal cord blood steroid profiles in relation to fetal sex and mode of delivery using temperature-dependent inclusion chromatography and principal component analysis (PCA). J Chromatogr B Analyt Technol Biomed Life Sci (2007) 855(2):249–54 10.1016/j.jchromb.2007.05.041 17625993

[41] Hill M Pařízek A Kancheva R Dušková M Velíková M Kříž L Steroid metabolome in plasma from the umbilical artery, umbilical vein, maternal cubital vein and in amniotic fluid in normal and preterm labor. J Steroid Biochem Mol Biol (2010) 121(3–5):594–610 10.1016/j.jsbmb.2009.10.012 19897033

[42] Dawood MY Saxena BB Testosterone and dihydrotestosterone in maternal and cord blood and in amniotic fluid. Am J Obstet Gynecol (1977) 129(1):37–42 900166 10.1016/0002-9378(77)90815-8

[43] Gemer O Sevillia J Zalis J Segal S Umbilical cord androgens in infants of diabetic mothers. Arch Gynecol Obstet (1997) 259(3):139–41 10.1007/BF02505322 9187466

[44] Hickey M Doherty DA Hart R Norman RJ Mattes E Atkinson HC Maternal and umbilical cord androgen concentrations do not predict digit ratio (2D:4D) in girls: a prospective cohort study. Psychoneuroendocrinology (2010) 35(8):1235–44 10.1016/j.psyneuen.2010.02.013 20299156

[45] Laatikainen TJ Pelkonen JM Pesonen AK Steroid concentrations in placenta and fetal membranes at elective caesarean section and after spontaneous labour. Placenta (1982) 3(3):319–24 10.1016/S0143-4004(82)80008-8 7134199

[46] Lagiou P Samoli E Okulicz W Xu B Lagiou A Lipworth L Maternal and cord blood hormone levels in the United States and china and the intrauterine origin of breast cancer. Ann Oncol (2011) 22(5):1102–8 10.1093/annonc/mdq565 20943596

[47] Mathur RS Landgrebe S Moody LO Powell S Williamson HO Plasma steroid concentrations in maternal and umbilical circulation after spontaneous onset of labor. J Clin Endocrinol Metab (1980) 51(6):1235–8 10.1210/jcem-51-6-1235 7440692

[48] Nagata C Iwasa S Shiraki M Sahashi Y Shimizu H Association of maternal fat and alcohol intake with maternal and umbilical hormone levels and birth weight. Cancer Sci (2007) 98(6):869–73 10.1111/j.1349-7006.2007.00464.x 17428259 PMC11158880

[49] Rohrmann S Sutcliffe CG Bienstock JL Monsegue D Akereyeni F Bradwin G Racial variation in sex steroid hormones and the insulin-like growth factor axis in umbilical cord blood of male neonates. Cancer Epidemiol Biomarkers Prevent (2009) 18(5):1484–91 10.1158/1055-9965.EPI-08-0817 PMC3012385 19423525

[50] Savarese T Strohsnitter W Low H Liu Q Baik I Okulicz W Correlation of umbilical cord blood hormones and growth factors with stem cell potential: implications for the prenatal origin of breast cancer hypothesis. Breast Cancer Res (2007) 9(3):1–10 10.1186/bcr1674 17501995 PMC1929091

[51] Simmons D Interrelation between umbilical cord serum sex hormones, sex hormone-binding globulin, insulin-like growth factor i, and insulin in neonates from normal pregnancies and pregnancies complicated by diabetes. J Clin Endocrinol Metab (1995) 80(7):2217–21 10.1210/jc.80.7.2217 7608282

[52] Troisi R Hoover RN Thadhani R Hsieh CC Sluss P Ballard-Barbash R Maternal, prenatal and perinatal characteristics and first trimester maternal serum hormone concentrations. Br J Cancer (2008) 99(7):1161–4 10.1038/sj.bjc.6604639 18766187 PMC2567091

[53] Troisi R Lagiou P Trichopoulos D Xu B Chie L Stanczyk FZ Cord serum estrogens, androgens, insulin-like growth factor-i, and insulin-like growth factor binding protein-3 in Chinese and U.S. Caucasian neonates. Cancer Epidemiol Biomarkers Prev (2008) 17(1):224–31 10.1158/1055-9965.EPI-07-0536 18199728

[54] Troisi R Potischman N Johnson CN Roberts JM Lykins D Harger G Estrogen and androgen concentrations are not lower in the umbilical cord serum of pre-eclamptic pregnancies. Cancer Epidemiol Biomarkers Prev (2003) 12(11):1268–70 14652293

[55] Troisi R Potischman N Roberts JM Ness R Crombleholme W Lykins D Maternal serum oestrogen and androgen concentrations in preeclamptic and uncomplicated pregnancies. Int J Epidemiol (2003) 32(3):455–60 10.1093/ije/dyg094 12777436

[56] Rosner W Auchus RJ Azziz R Sluss PM Raff H Utility, limitations, and pitfalls in measuring testosterone: an endocrine society position statement. J Clin Endocrinol Metab (2007) 92(2):405–13 10.1210/jc.2006-1864 17090633

[57] Stanczyk FZ Measurement of androgens in women. Paper Presented at the Seminars in Reproductive Medicine New York (2006). 10.1055/s-2006-939566 16633981

[58] Demers LM Androgen deficiency in women; role of accurate testosterone measurements. Maturitas (2010) 67(1):39–45 10.1016/j.maturitas.2010.04.019 20493647

[59] Stanczyk FZ Cho MM Endres DB Morrison JL Patel S Paulson RJ Limitations of direct estradiol and testosterone immunoassay kits. Steroids (2003) 68(14):1173–8 10.1016/j.steroids.2003.08.012 14643879

[60] Krogh C Cohen AS Basit S Hougaard DM Biggar RJ Wohlfahrt J Testosterone levels in umbilical-cord blood and risk of pyloric stenosis. Pediatrics (2011) 127(1):e197–201 10.1542/peds.2010-2127 21172998

[61] Soldin OP Guo T Weiderpass E Tractenberg RE Hilakivi-Clarke L Soldin SJ Steroid hormone levels in pregnancy and 1 year postpartum using isotope dilution tandem mass spectrometry. Fertil Steril (2005) 84(3):701–10 10.1016/j.fertnstert.2005.02.045 16169406 PMC3640374

[62] Stanczyk FZ Clarke NJ Advantages and challenges of mass spectrometry assays for steroid hormones. J Steroid Biochem Mol Biol (2010) 121(3–5):491–5 10.1016/j.jsbmb.2010.05.001 20470886

[63] Vicente FB Smith FA Sierra R Wang S Measurement of serum testosterone using high-performance liquid chromatography/tandem mass spectrometry. Clin Chem Lab Med (2006) 44:70 10.1515/CCLM.2006.014 16375589

[64] Jacklin CN Maccoby EE Doering CH Neonatal sex-steroid hormones and timidity in 6-18-month-old boys and girls. Dev Psychobiol (1983) 16(3):163–8 10.1002/dev.420160302 6873481

[65] Jacklin CN Maccoby EE Doering CH King DR Neonatal sex-steroid hormones and muscular strength of boys and girls in the first three years. Dev Psychobiol (1984) 17(3):301–10 10.1002/dev.420170309 6724145

[66] Jacklin CN Wilcox KT Maccoby EE Neonatal sex-steroid hormones and cognitive abilities at six years. Dev Psychobiol (1988) 21(6):567–74 10.1002/dev.420210607 3169381

[67] Hollier LP Mattes E Maybery MT Keelan JA Hickey M Whitehouse AJO The association between perinatal testosterone concentration and early vocabulary development: a prospective cohort study. Biol Psychol (2013) 92(2):212–5 10.1016/j.biopsycho.2012.10.016 23153707

[68] Whitehouse AJO Mattes E Maybery M Dissanayake C Sawyer M Jones R Perinatal testosterone exposure and autistic-like traits in the general population: a longitudinal pregnancy-cohort study. J Neurodev Disord (2012) 4(1):25 10.1186/1866-1955-4-25 23110806 PMC3500651

[69] Farrant BM Mattes E Keelan JA Hickey M Whitehouse AJO Fetal testosterone, socio-emotional engagement and language development. Infant Child Dev (2013) 22(2):119–32 10.1002/icd.1771

[70] Whitehouse AJO Mattes E Maybery MT Sawyer MG Jacoby P Keelan JA Sex-specific associations between umbilical cord blood testosterone levels and language delay in early childhood. J Child Psychol Psychiatry (2012) 53(7):726–34 10.1111/j.1469-7610.2011.02523.x 22276678

[71] Robinson M Whitehouse AJO Jacoby P Mattes E Sawyer MG Keelan JA Umbilical cord blood testosterone and childhood internalizing and externalizing behavior: a prospective study. PLoS One (2013) 8(4):e59991 10.1371/journal.pone.0059991 23573225 PMC3613417

[72] Rescorla L The language development survey: a screening tool for delayed language in toddlers. J Speech Hear Disord (1989) 54(4):587–99 2811339 10.1044/jshd.5404.587

[73] Berglund EVA Eriksson M Westerlund M Communicative skills in relation to gender, birth order, childcare and socioeconomic status in 18-month-old children. Scand J Psychol (2005) 46(6):485–91 10.1111/j.1467-9450.2005.00480.x 16277649

[74] Feldman HM Dollaghan CA Campbell TF Kurs-Lasky M Janosky JE Paradise JL Measurement properties of the Macarthur communicative development inventories at ages one and two years. Child Dev (2000) 71(2):310–22 10.1111/1467-8624.00146 10834466

[75] Zubrick SR Taylor CL Rice ML Slegers DW Late language emergence at 24 months: an epidemiological study of prevalence, predictors, and covariates. J Speech Lang Hear Res (2007) 50(6):1562–92 10.1044/1092-4388(2007/106) 18055773 PMC3521638

[76] Squires J Bricker D Potter A Infant/Child Monitoring Questionnaires Procedures Manual. Eugene: University of Oregon (1990).

[77] Knickmeyer RC Baron-Cohen S Raggatt P Taylor K Foetal testosterone, social relationships, and restricted interests in children. J Child Psychol Psychiatry (2005) 46(2):198–210 10.1111/j.1469-7610.2004.00349.x 15679528

[78] Fullard W McDevitt SC Carey WB Assessing temperament in one-to three-year-old children. J Pediat Psychol (1984) 9(2):205–17 10.1093/jpepsy/9.2.205 6470903

[79] Dunn LM Dunn LM Peabody Picture and Vocabulary Test. 4th ed Bloomington: Pearson (2007).

[80] Baron-Cohen S Wheelwright S Skinner R Martin J Clubley E The autism-spectrum quotient (aq): evidence from Asperger syndrome/high-functioning autism, males and females, scientists and mathematicians. J Autism Dev Disord (2001) 31(1):5–17 10.1023/A:1005653411471 11439754

[81] Auyeung B Taylor K Hackett G Baron-Cohen S Foetal testosterone and autistic traits in 18 to 24-month-old children. Mol Autism (2010) 1(1):11 10.1186/2040-2392-1-11 20678186 PMC2916006

[82] Lutchmaya S Baron-Cohen S Raggatt P Foetal testosterone and eye contact in 12-month-old human infants. Infant Behav Dev (2002) 25(3):327–35 10.1016/S0163-6383(02)00094-2

[83] Auyeung B Baron-Cohen S Ashwin E Knickmeyer R Taylor K Hackett G Fetal testosterone and autistic traits. Br J Psychol (2009) 100:1–22 10.1348/000712608X311731 18547459

[84] Achenbach TM Manual for the Child Behaviour Checklist/4-18. Burlington: University of Vermont (1991).

[85] Achenbach TM Edelbrock C Howell CT Empirically based assessment of the behavioral/emotional problems of 2- and 3-year-old children. J Abnorm Child Psychol (1987) 15(4):629–50 10.1007/BF00917246 3437096

[86] Roselli CE Estill CT Stadelman HL Meaker M Stormshak F Separate critical periods exist for testosterone-induced differentiation of the brain and genitals in sheep. Endocrinology (2011) 152(6):2409–15 10.1210/en.2010-1445 21447635 PMC3206706

